# Frequency of coreceptor tropism in PBMC samples from HIV-1 recently infected blood donors by massively parallel sequencing: the REDS II study

**DOI:** 10.1186/s12985-015-0307-3

**Published:** 2015-05-14

**Authors:** Rodrigo Pessôa, Ester C Sabino, Sabri S Sanabani

**Affiliations:** Department of Pathology, Hospital das Clínicas, School of Medicine, University of São Paulo, São Paulo, Brazil; Department of Infectious Disease/Institute of Tropical Medicine, University of São Paulo, Sao Paulo, Brazil; Medicina Instituto de Medicina Tropical de São Paulo, LIM 52 - Av. Dr. Enéas Carvalho de Aguiar, 470 - 2° andar - Cerqueira Cesar, 05403-000 Sao Paulo, SP Brazil

## Abstract

**Background:**

The interaction of HIV-1 and target cells involves sequential binding of the viral gp120 Env protein to the CD4 receptor and a chemokine co-receptor (either CCR5 or CXCR4). CCR5 antagonists have proved to be an effective salvage therapy in patients with CCR5 using variants (R5) but not with variants capable of using CXCR4 (×4) phenotype. Thus, it is critically important to determine cellular tropism of a country’s circulating HIV strains to guide a management decision to improve treatment outcome. In this study, we report the prevalence of R5 and ×4 HIV strains in 45 proviral DNA massively parallel sequencing “MPS” data from recently infected Brazilian blood donors.

**Methods:**

The MPS data encompassing the tropism-related V3 loop region of the HIV‐1 *env* gene was extracted from our recently published HIV-1 genomes sequenced by a paired-end protocol (Illumina). HIV‐1 tropism was inferred using Geno2pheno_[coreceptor]_ algorithm (3.5 % false-positive rate). V3 net charge and 11/25 rules were also used for coreceptor prediction.

**Results:**

Among the 45 samples for which tropism were determined, 39 were exclusively R5 variants, 5 ×4 variants, and one dual-tropic or mixed (D/M) populations of R5 and ×4 viruses, corresponding to 86.7, 11.1 and 2.2 %, respectively. Thus, the proportion of all blood donors that harbor CXCR4-using virus was 13.3 % including individuals with D/M-tropic viruses.

**Conclusions:**

The presence of CCR5-tropic variants in more than 85 % of our cohort of antiretroviral-naïve blood donors with recent HIV-1 infection indicates a potential benefit of CCR5 antagonists as a therapeutic option in Brazil. Therefore, determination of viral co-receptor tropism is an important diagnostic prerequisite.

## Background

The interaction of human immunodeficiency virus type 1(HIV-1) and target cells involves sequential binding of the viral gp120 Env protein to the CD4 receptor and a chemokine co-receptor [1]. The high selection pressures exerted on the viral gp120 molecule explain why the HIV-1 viral populations have very high genetic diversity. Early after infection, HIV-1 variants are largely or exclusively bind to β-chemokine co-receptor CCR5 in conjunction with CD4 molecules; such variants are termed ‘R5 viruses’ [2]. These viruses are non-syncytium-inducing isolates and do not replicate in T-cell lines, but replicate well in macrophages and are known as macrophage tropic strains. T-cell line-tropic HIV-1 viruses using another α-chemokine receptor, CXCR4, called ×4 variants while dual tropic or mixed (D/M) populations can interact with both CCR5 and CXCR4 coreceptors in the fusion process [3]. The emergence of ×4 viruses and D/M isolates generally occur at the later stage of infection and are consistently associated with increased severity of disease in roughly half of all persons infected with HIV [4–6]. Currently, there is much interest in determining coreceptor tropism before initiating treatment with the CCR5 coreceptor blocker maraviroc which has exclusive activity against R5 viruses [7, 8]. HIV-1 tropism may be determined by phenotypic or genotypic assays. Phenotypic testing assess the ability of pseudoviruses carrying the entire cloned *env* gene from a patient’s virus to infect CCR5 or CXCR4 reporter cell-lines that also express CD4 molecules [9]. Although this approach has demonstrated good sensitivity and correlates well with clinical outcome [7], phenotypic testing are complex to perform, prohibitively expensive, and time-consuming. It may also be inferred genotypically from the 35-amino-acid V3 loop region of the viral envelope protein, gp120 sequence [10]. Emerging data from several studies indicate that genotypic approach has several advantages over the phenotypic assay that include a low cost, simpler technical demands, faster turnaround time, and more suitable to a large series compared with phenotypic tropism testing [11, 12]. Moreover, genotypic predictors proved to be highly concordant with phenotype data and can reliably be used to determine viral tropism particularly in treatment-experienced patients [8, 12, 13]. Previous studies generally indicated that CXCR4-using viruses carry positively charged amino acids in the V3 loop, while CCR5-tropic viruses do not [14, 15]. In a clinical setting, detection of ×4 variants at low concentrations is considered important because they may potentially emerge during therapy with a CCR5 antagonist. To improve the laboratory detection sensitivity of ×4 minority species in aviremic patients, the European Consensus Group guidelines recommended generation of sequences through independent triplicate PCR amplification and/or by deep sequencing technology [12, 16].

Despite the fact that Maraviroc has been used in Brazil since 2007, few data are available about its efficacy during routine use. Recently, Alencar *et al.* [17] found that 27.5 % of samples from patients failing previous antiretroviral therapy harbored one or more mutations that confer some degree of susceptibility to maraviroc. In another study, Araújo and coworkers [18] reported that most of the resistance-associated mutations in ARV-naïve patients occur in subtype C compared with subtype B strains.

Here, we report the prevalence of R5, ×4, and D/M variants of HIV-1 from massively parallel sequencing “MPS” proviral data generated during the early phase of HIV-1 infection in a group of first-time Brazilian blood donors. Although plasma HIV-1 RNA has been widely used to determine the viral tropism the proviral PBMC DNA sequence can contain a variety of multiple archived genomes that are not present in plasma. This, combined with the stability of DNA compared with RNA, and the fact that HIV DNA recovered from the proviral compartment can reliably be used as an alternative to RNA tropism testing [19–22] influenced our decision to use proviral DNA in this study.

## Methods

Previously, we had described the genetic diversity of HIV-1 using partial (*n* = 6) and near full-length genomes (NFLG) sequence (*n* = 39) of human immunodeficiency virus Type 1 provirus deep sequencing data from recently infected donors at four blood centers participating in the Retrovirus Epidemiology Donor Study (REDS-II) International Program in Brazil [23]. Samples were classified by less-sensitive (LS) or “detuned” enzyme immunoassay (Vironostika HIV-1 MicroElisa; bioMérieux, Durham, NC) or an LS chemiluminescent immunoassay (Vitros HIV-1/2 Assay; Ortho Diagnostics, Rochester, NY) as recently infected at the time of donation based on antibody levels consistent with recent seroconversion (infected for <170 days) as previously described [24]. None of the participants received antiviral treatment before. All study subjects provided written informed consent. The study was approved by the local ethical review committee of participating institutions as well as the REDS-II collaborating centers (Blood Systems Research Institute/University of California at San Francisco, San Francisco, CA) and data coordinating center (Westat, Inc.) in the United States.

### Extraction of reads spanning the V3 region from HIV MPS

In this study, a sub-library of the *env* V3 population sequence derived from each sample was created by mapping the raw MPS short reads to their corresponding V3 consensus sequence (Sequences positions: 210 to 315 [GenBank accession no. K03455] in standard reference HXB2) using the CLC Genomics Workbench version 7.0.4 (CLC Bio, Aarhus, Denmark). To avoid artificial generation of *in silico* chimeras through assembly and to evade inflating the diversity estimates of the V3 region, the analysis was restricted to individual paired-end reads that encompass the complete V3 region from each dataset. The reads were aligned, truncated and translated for genotyping. Prior to the determination of viral tropism, the MPS data were filtered out by the presence of frame shifts, stop codons, and base-call ambiguity.

### Determination of HIV-1 coreceptor tropism

HIV-1 co-receptor tropism was assessed from the filtered V3 MPS data using the new prediction tool geno2pheno-C_NGS-Sanger implemented in the Geno2Pheno [coreceptor] (http://coreceptor.bioinf.mpi-inf.mpg.de/), which uses support vector machine technology. To minimize the number of false predictions of CXCR4 tropic sequences as CCR5 tropic, tropism was inferred using cutoffs optimized and validated in the maraviroc treatment-experienced trials and A4001029 clinical trials [16, 25]. Therefore, ×4 or D/M viruses (non-R5) were reported positive if their sequences had a prediction FPR result of ≤ 3.5 % (3.5 % probability of classifying an R5 virus falsely as ×4) or the 11/25 rule [26] predicted a ×4 virus, otherwise, they were considered CCR5-tropic viruses. The detection threshold of minor ×4 variants varied according to the number of extracted full-length reads of V3 for each sample. Moreover, the overall net charge (NC) of V3 amino acid (R + K − D − E) were assigned to each sequence fragment to predict HIV-1 tropism [27, 28]. Sequences with NC values <5.0 were classified as R5, whereas sequences with NC values ≥ 5.0 were classified as ×4.

### Nucleotide distance analysis

The intra-host viral genetic diversities of the V3 nucleotide sequences were computed from all available deep sequences in each clinical sample using the maximum composite likelihood in MEGA version 6 [29].

### Nucleotide sequence accession numbers

The sequencing data have been uploaded to zenodo https://zenodo.org/ (DOI: 10.5281/zenodo.14666).

## Results

The near full-length genomes and/or larger fragments of the 45 V3 MPS data used in this study have recently been described for their genetic variability [23]. This analysis indicated that 28 (62.2 %) were subtype B sequences, 11 (24.4 %) BF1 recombinants, 2 (4.4 %) BC recombinants, 1 (2.2 %) were BC and BCF1 each, 1 (2.2 %) CRF45_cpx, and 1 (2.2 %) were the newly described CRF70_BF1 [30]. The tropism predictions, FPR values, and V3 net charges are shown in Table 1. The datasets of the V3 sequence extracted from the NFLGs ranged from 7 sequences in patient 10BR_SP048 to 14026 sequences in patient 10BR_PE091. After removal of scaffolding reads not covering the complete V3 region, the coverage dropped from 282 to one read in subject 10BR_MG029. Overall, 68.9 % of the samples showed a V3 loop region covered by more than 100 sequencing reads. All MPS reads from the 45 proviral samples in which a complete V3-loop sequence was found were submitted to the geno2pheno [coreceptor] prediction tool. This analysis revealed virus populations with a pure R5 and ×4 phenotype in 39 (86.7 %) and 5 (11.1 %) blood donors, respectively. The presence of D/M-tropic sequences was found in one (2.1 %) subject. Thus, the proportion of all blood donors that harbor CXCR4-using virus was 13.2 % including the donor with D/M-tropic viruses. According to the NC, 66.6 % of sequences predicted as ×4 by geno2pheno showed NC values ≥ 5; similarly, 95 % of sequences predicted as R5 by geno2pheno had NC values below 5. Regarding the D/M viruses, the V3 domains sequences displayed NC < 5.

**Table 1:** Genetic subtype, coreceptor usage, net charge and sequence coverage across V3 region in PBMC for the HIV-1 isolates

Sample ID	Subtype based on NFLG^1^	geno2pheno (3.5 % FPR^2^)	Coverage including all short reads	Overall mean Div.%	Coverage of the V3 reads after filteration	Subtype based on the V3 sequence	Number of seq. with charges <5/≥5
10BR_001PE	B	×4	322	1.1	132	B	06/126
10BR_003MG	BF1	R5	9985	0.7	532	F1	531/01
10BR_003SP	B	R5	876	1.4	296	B	270/26
10BR_007MG	B	R5	80	1.3	4	B	04/00
10BR_008SP	B	R5	655	0.7	120	B	119/1
10BR_009SP	B	R5	1031	1.0	290	B	271/19
10BR_010MG	BCF1	R5	13756	1.0	763	F1	736/27
10BR_010PE	B	R5	1103	0.8	345	B	330/15
10BR_011SP	CRF45_cpx	R5	24	0.8	10	A1	10/00
10BR_014SP	B	R5	404	0.7	176	B	174/02
10BR_015RJ	BF1	R5	3687	1.4	818	F1	813/05
10BR_017SP	BF1	R5	494	0.4	8	F1	08/00
10BR_017MG	BF1	R5	31	0.7	108	F1	108/00
10BR_018PE	BC	R5	48	0.0	13	B	13/00
10BR_021SP	B	R5	838	1.0	289	B	288/01
10BR_023RJ	BF1	R5/×4	446	1.5	248	F1	248/00
10BR_026RJ	BF1	R5	3281	1.1	888	B	48/840
10BR_029MG	B	×4	282	0.0	1	B	00/01
10BR_029PE	BCF1	×4	909	0.9	183	F1	06/177
10BR_032RJ	B	R5	1732	2.0	733	B	733/00
10BR_034MG	BF1	R5	844	0.7	51	B	51/00
10BR_034PE	B	R5	679	0.9	369	B	350/19
10BR_035MG	B	R5	851	1.2	469	B	462/07
10BR_038SP	B	R5	2247	1.8	352	B	338/15
10BR_041RJ	BF1	R5	342	0.8	160	F1	147/13
10BR_042SP	B	R5	1269	2.0	293	B	279/14
10BR_043SP	B	×4	65	1.7	26	B	006/2020
10BR_045SP	B	R5	137	0.0	8	B	08/00
10BR_046RJ	CRF70_BF1	R5	112	0.4	32	B	32/00
10BR_046SP	CF1	R5	2830	2.0	278	C	273/5
10BR_048SP	BF1	R5	7	0.0	5	B	05/00
10BR_049SP	B	R5	632	3.5	44	B	44/0
10BR_050RJ	B	R5	1695	2.5	341	B	335/06
10BR_050SP	B	R5	1067	2.2	47	B	35/12
10BR_051RJ	B	×4	4301	0.5	482	B	474/08
10BR_053PE	B	R5	2480	2.0	784	B	750/34
10BR_053RJ	BF1	R5	3718	0.9	2011	F1	1888/123
10BR_054RJ	B	R5	2173	0.9	745	B	655/90
10BR_055SP	B	R5	1393	1.5	59	B	59/00
10BR_056PE	B	R5	551	0.5	10	B	00/10
10BR_057SP	BF1	R5	2704	0.6	1322	F1	1319/3
10BR_091PE	B	R5	14026	0.8	2165	B	2161/4
10BR_097PE	B	R5	3773	3.3	697	B	675/22
10BR_098PE	B	R5	10215	5.3	1456	B	1311/145
10BR_100PE	B	R5	6060	3.7	614	B	613/01

^1^Near Full-Length genomes, 2 False positive rate

**Figure 1:**
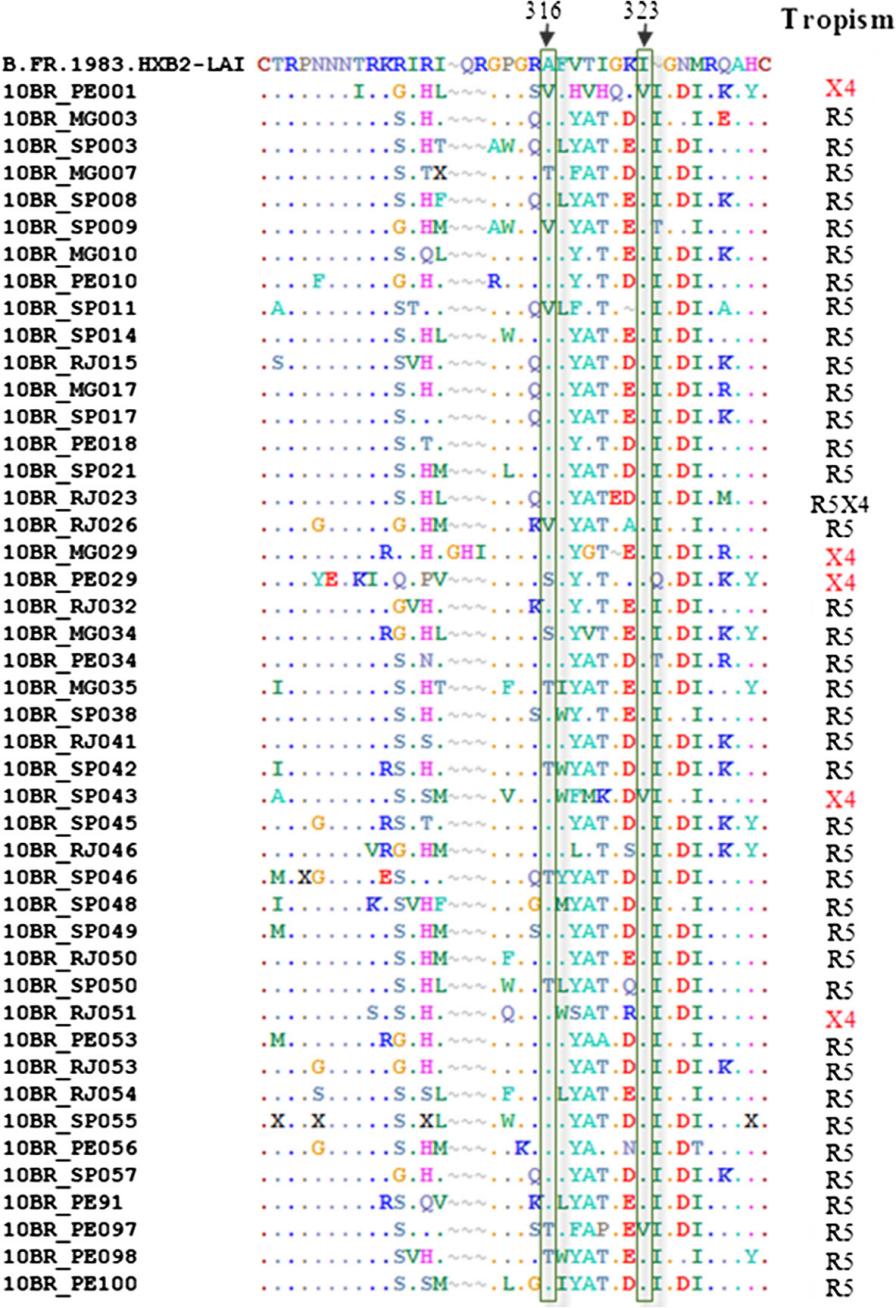
Amino acid alignment of V3 sequences. The consensus sequence from each sample was generated and aligned with HXB2 (Accession number: K03455). Amino acid positions appear at the top of alignment. Position 316 and 323, corresponding to the third position of the codon encoding the amino acid at position 11 and 25 of the V3 loop. Dots indicate identities whereas tildes indicate deletions

The V3 consensus sequences from the 45 samples were aligned and investigated for the presences of the GWGR motif in the V3 loop, a feature commonly observed on the Brazilian B subtype samples (Fig. 1). The tetrapeptide GWGR\AWGR motif in the V3 loop apex sequence was observed in 5 (11.1 %) donors infected with pure subtype B and all had predicted R5 viruses. The aligned sequences were also analyzed for the presence of A316T and I323V resistance-conferring point mutations to maraviroc in R5 and D/M viruses The A316T substitution was detected in 7 (17.9 %) donors, whereas the I323V substitution was detected in only three (7.7 %) subjects; both mutations have been shown to confer maraviroc partial resistance [31]. Phenotypic assays are necessary to confirm the influence of this mutation to maraviroc susceptibility. Since maraviroc was used in Brazil after 2007 on therapy-failure patients, high rate in treatment-naïve samples may be related to the transmission of maraviroc-resistant variants from patients with treatment experience.

## Discussion

In this study, we sought to estimate the prevalence of coreceptor tropism of the archived strains at the time of primary infection using a total of 45 MPS data from HIV-1 recently infected Brazilian first-time blood donors. We found 6 ×4 strains (13.3 %) including the D/M tropic populations (*n* = 1) and 39 exclusive R5 variants (86.7 %). The prevalence found for ×4 DNA was similar to that reported 16.4 %–17.2 % of ×4 and D/M-tropic strains in recent HIV-1 seroconverter Spanish subjects [32, 33]. These results were also comparable to those of Frange *et al.* [34] who reported a relatively high frequency (15.9 %) of ×4 and D/M virus in 390 HIV-1 subtype-B infected patients diagnosed at the time of primary infection. Our results were also consistent with those of a previous study in drug-naive chronically HIV-infected individuals [35, 36] and in suppressed patients with a shorter history of viremia suppression [37–39]. In contrast, our prevalence estimates of CXCR4-using viruses is higher than those found in 126 recently infected men having sex with men in the USA study of 3.2 % [40] and less than the rate of prevalence reported in our previous study in recently infected Brazilian subjects (30.2 %) [41]. Factors that might have contributed to the differences observed were the sample size, type of samples, the sequencing method, the test replications, the FPR cutoff, and prediction algorithms used.

The relatively high rate of CXCR4-using viruses in this study may be explained by the application of deep sequencing technology which has improved the prediction of HIV tropism as has been reported in previous studies [16, 42, 43]. Using our approach, we were able to detect one ×4 variant that existed as a mixture along with R5 isolates in subject 10BR_023RJ. If standard population-based sequencing data had been used alone, we would have not been able to detect the ×4 variants in that subject as it presents in 3.2 % of the viral population. The detection of dual or mixed R5×4 viral strains from sample collected at the earliest time point could be the result of concomitant transmission of multiple variants, or successive infections within a short timeframe. The assumption of direct transmission of the mixed variants in subject 10BR_023RJ do not support the hypothesis of gatekeeping event that almost always selects for transmission of R5 over ×4 HIV-1 strains [44]. Indeed, there is no convincing evidence has yet been published to proof the lower transmissibility of ×4 viruses but available data support the idea that R5 or D/M infections could result from a stochastic process [45, 46].

This relatively high prevalence should seriously be considered when decisions are made about initial regimens for therapy-naive individuals, and HIV-1 coreceptor usage should be screened before initiation of any chemokine receptor CCR5 antagonists in clinical settings. These suggestions are in agreement with the conclusions of Frange *et al.* [34] that noted that ×4/DM strains can fuel the cellular HIV-1 reservoir leading to viral persistence over a long period complicating future therapeutic options, including CCR5 antagonists.

One of the major limitations beside the small sample size of this study is that the assessment of HIV tropism was limited to sequence- based algorithms rather than using phenotypic methods. Although phenotypic assays still have an edge over genotypic methods, genotypic predictors prove to be highly concordant with phenotype data and can reliably be used to determine viral tropism with better results in PBMC than in plasma samples [47]. In this study, we used geno2pheno because it allows for an adjustable cutoff, and it can determine HIV-1 co-receptor usage in all viral genotypes. This method has shown a similar performance to the Trofile phenotypic assay, the most often used tropism method [48]. Moreover, the method has been shown to achieve higher sensitivity while retaining high level of specificity when compared with the performance of different algorithms [49, 50]. In some samples there were little or no sequencing coverage in the V3 region and this may have biased the results of this study.

## Conclusions

Although the sample size is small and not representative, our findings add further support to the previous studies and show that ×4 variants may be frequently found at a relatively high proportion in early infected subjects. More studies with large samples size are needed to replicate our findings and to explore the clinical relevance of the variants with predicted usage of CXCR4 present in the light of both clinical progression and therapeutic approach. In conclusion, while suggesting CCR5 antagonists (maraviroc) as useful therapeutic approach, it has to be noted as a caution, that maraviroc resistant R5 strains described in present study were isolated from non-treated, recently HIV-1 infected individuals.

## Abbreviations

HIV-1: Human immunodeficiency virus type 1
CXCR4: chemokine receptor type 4
CCR5: C-C chemokine receptor type 5
×4: CXCR4-using virus
R5: CCR5-using virus
D/M: Dual-mixed virus
FPR: False positive rate
MPS: massively parallel sequencing
PBMCs: Peripheral blood mononuclear cells
NC: Net charge
